# Opposing Effects of the Angiopoietins on the Thrombin-Induced Permeability of Human Pulmonary Microvascular Endothelial Cells

**DOI:** 10.1371/journal.pone.0023448

**Published:** 2011-08-15

**Authors:** Melanie van der Heijden, Geerten P. van Nieuw Amerongen, Jan van Bezu, Marinus A. Paul, A. B. Johan Groeneveld, Victor W. M. van Hinsbergh

**Affiliations:** 1 Department of Intensive Care, Institute for Cardiovascular Research, VU University Medical Centre, Amsterdam, The Netherlands; 2 Department of Physiology, Institute for Cardiovascular Research, VU University Medical Centre, Amsterdam, The Netherlands; 3 Department of Cardiothoracic Surgery, VU University Medical Centre, Amsterdam, The Netherlands; University of Illinois at Chicago, United States of America

## Abstract

**Background:**

Angiopoietin-2 (Ang-2) is associated with lung injury in ALI/ARDS. As endothelial activation by thrombin plays a role in the permeability of acute lung injury and Ang-2 may modulate the kinetics of thrombin-induced permeability by impairing the organization of vascular endothelial (VE-)cadherin, and affecting small Rho GTPases in human pulmonary microvascular endothelial cells (HPMVECs), we hypothesized that Ang-2 acts as a sensitizer of thrombin-induced hyperpermeability of HPMVECs, opposed by Ang-1.

**Methodology/Principal Findings:**

Permeability was assessed by measuring macromolecule passage and transendothelial electrical resistance (TEER). Angiopoietins did not affect basal permeability. Nevertheless, they had opposing effects on the thrombin-induced permeability, in particular in the initial phase. Ang-2 enhanced the initial permeability increase (passage, P = 0.010; TEER, P = 0.021) in parallel with impairment of VE-cadherin organization without affecting VE-cadherin Tyr685 phosphorylation or increasing RhoA activity. Ang-2 also increased intercellular gap formation. Ang-1 preincubation increased Rac1 activity, enforced the VE-cadherin organization, reduced the initial thrombin-induced permeability (TEER, P = 0.027), while Rac1 activity simultaneously normalized, and reduced RhoA activity at 15 min thrombin exposure (P = 0.039), but not at earlier time points. The simultaneous presence of Ang-2 largely prevented the effect of Ang-1 on TEER and macromolecule passage.

**Conclusions/Significance:**

Ang-1 attenuated thrombin-induced permeability, which involved initial Rac1 activation-enforced cell-cell junctions, and later RhoA inhibition. In addition to antagonizing Ang-1, Ang-2 had also a direct effect itself. Ang-2 sensitized the initial thrombin-induced permeability accompanied by destabilization of VE-cadherin junctions and increased gap formation, in the absence of increased RhoA activity.

## Introduction

Excessive and sustained activation of the pulmonary endothelium is central in the pathogenesis of the pulmonary inflammation and permeability of the life-threatening syndromes acute lung injury (ALI) and acute respiratory distress syndrome (ARDS) [1]. In experimental models of ALI, the angiopoietin-Tie2 receptor system modulates the responsiveness of the pulmonary endothelium [2]–[5]. Indeed, angiopoietin-1 (Ang-1) reduced pulmonary inflammation and permeability [2]–[8], whereas its antagonist angiopoietin-2 (Ang-2) sensitized the pulmonary endothelium to inflammatory stimuli [9], [10]. Consistent with these experimental data, circulating Ang-2 related to vascular permeability and pulmonary dysfunction in critically ill patients [11]–[13].

Activation of coagulation is both a consequence and a contributor to ALI/ARDS, since the pro-coagulant state results in intra-alveolar fibrin deposition, which enhances inflammation [14]. Furthermore, the pro-coagulant protein thrombin is massively generated during ALI [15] and has direct effects on vascular permeability via intercellular gap formation [16]–[18]. Interestingly, Ang-1 attenuated the thrombin-induced permeability in human umbilical vein and bovine pulmonary endothelial cells [19]–[21]. Nevertheless, the effect of Ang-2 on the thrombin-response has not been studied. In addition, ALI/ARDS was not appropriately modeled using those cell types, since endothelial cells from different vascular beds display remarkable heterogeneity in structure and function [22]–[24]. Therefore, it remains to be investigated in an in vitro model of ALI using human pulmonary microvascular endothelial cells (HPMVECs), whether Ang-2 modulates the thrombin-induced permeability and which pathways are involved.

The effect of the Ang-2 on the kinetics of the thrombin response is of specific interest, since different molecular mechanisms play a role during the distinct phases of the response [16]. Indeed, during the initial rapid increase in permeability after thrombin stimulation, disruption of adherence junctions between cells, amongst others due to reduced Rac1 activity [25] and subsequently RhoA-mediated endothelial contraction [26], play a role [16], [27]. When the maximum increase in permeability is reached, both disruption of adherence junctions and endothelial contraction play a role [16], [26].

For the current study it was hypothesized that Ang-2 increases basal and thrombin-induced permeability of HPMVECs by impairing vascular endothelial cadherin (VE-cadherin) junctional organization in part via reduced Rac1 and increased RhoA activity. Since Ang-1 has been extensively studied before, Ang-2 data were compared to Ang-1 data.

## Materials and Methods

### Isolation and culture of HPMVECs

HPMVECs were isolated as previously described (supporting information Text S1) [24]. Five days after isolation, HPMVECs formed small islands in culture. Nine days after isolation, HPMVEC islands were confluent. After a second magnetic separation of HPMVECs and non-endothelial cells, the culture showed a purity of >99% as confirmed by the presence of endothelial cell markers VE-cadherin, CD31, von Willebrand factor (VWF), Tie2 and endothelial nitric oxide synthase (eNOS) and the absence of smooth muscle cell (SMC) marker α-actin and epithelial cell marker pancytokeratin (supporting information Figure S1). HPMVECs had a relatively low basal permeability, compared to human umbilical vein endothelial cells (HUVECs, basal transendothelial electrical resistance (TEER) 41.3±3.0 Ω·cm^2^ vs. 27.6±3.8 Ω·cm^2^, P = 0.014).

### Determination of the angiopoietin release of HPMVECs

Microvascular endothelial cell medium-2 (EGM-2-MV, Lonza, Basel, Switzerland) was put on a confluent HPMVEC monolayer for 0, 24, 48 or 72 hours. At each time point, medium was collected and Ang-2 and Ang-1 concentrations were measured in duplicate using the human Ang-2 and Ang-1 DuoSet ELISA Development kits (R&D systems, Minneapolis, Minneapolis, USA) according to the manufacturers protocol. Experiments were also performed in cells stimulated with 0.1 U/ml thrombin to measure endogenous Ang-2 release.

### Determination of the endothelial permeability

In vitro, endothelial permeability can be evaluated by culturing cells on porous filters and subsequent assessment of the horse radish peroxidase (HRP, 40 kDa) passage or the transendothelial ion-flux via cell-cell and cell-matrix contacts as indicated by the TEER [28], [29]. Since the relationship between the macromolecule flux and the TEER is non-linear and the passage is size-dependent [28]–[30] both were assessed.

The transfer of HRP over the HPMVEC monolayer was measured as previously described [26]–[28]. In brief, confluent monolayers of HPMVECs (passage 3–7) were harvested with trypsin/ethyleendiaminetetraacetic acid (EDTA) and seeded in high density on fibronectin-coated polycarbonate filters of the Transwell system (0.33 cm^2^, pore size 3.0 µm, Corning Incorporated Life Sciences, Lowell, Massachusetts, USA). EGM-2-MV medium with 5% fetal bovine serum was renewed every other day. Monolayers were used 5 days after seeding. One hour before the start of the experiment, monolayers were serum starved in 1% human serum albumin (HSA, Sanquin, Amsterdam, The Netherlands) in endothelial cell basal medium-2 (EBM-2, Lonza). At the start of the experiment (time = −30 min), HRP (5 µg/ml) with or without Ang-2 (400 ng/ml), Ang-1 (400 ng/ml) or the combination in 1% HSA was added to the upper compartment of the Transwell system. At time = 0 min, the first sample from the lower compartment was taken and an equal volume of 1% HSA was added to the lower compartment. Immediately after the first sample was taken, thrombin (0.2 U/ml, Sigma, St. Louis, Missouri, USA) was added to the upper compartment. The other samples from the lower compartment were taken at time = 15, 30, 90 and 210 min. At the end of the experiment, a sample was taken from the upper compartment. Filters were kept at 37°C under 5% CO_2_/95% air atmosphere during the experiment. The concentration of HRP was derived from the HRP activity in each sample with peroxide and tetramethylbenzidine as substrate. The basal HRP passage at t = 0 min (% of HRP input/hour) and the initial and the prolonged HRP passage rate from 0–15 and from 30–90 min after thrombin addition, respectively, were calculated.

The TEER was measured as previously described [28]. HPMVECs were seeded on the Transwell system as described above. After serum starvation, Ang-2 (400 ng/ml), Ang-1 (400 ng/ml) or the combination was added to the upper compartment of the filter and the filter was placed in the TEER apparatus (t = −30 min). The filter was left in the apparatus for 10 min to acclimatize before recording started. An alternating current (50 µA, 2 pulses per min) was passed across the monolayer by two source electrodes. The potential difference across the monolayer was measured by two detecting electrodes. Thrombin (0.2 U/ml) was added to the upper compartment of the filter at t = 0 min. The recording continued until t = 40 min. Filters were kept at 37°C under 100% air atmosphere during the experiment. The electrical resistance was calculated by Ohm's law and expressed in Ω•cm^2^. Electrical resistance of the filter without an endothelial monolayer (8 Ω•cm^2^) was subtracted from all measured values. The absolute basal TEER at t = 0 min, the thrombin-induced decrease in TEER per min from 0–10 min after thrombin-stimulation and the prolonged maximum thrombin-induced decrease in TEER were calculated.

### Analysis of Tie2 and vascular endothelial cadherin (VE-cadherin) phosphorylation

After serum starvation, Ang-2 (400 ng/ml), Ang-1 (200 ng/ml) or the combination were added to the well chamber. Cells were lysed with lysis buffer (20 mM Tris/HCl pH 8.0, 150 mM NaCl, 90 mM KCl, 2 mM EDTA/NaOH pH 8.0, igepal (1∶200), triton X-100 (1∶200), 1 mM Na_3_VO_4_, 10 mM NaF, protease inhibitors (1∶100), phosphatase inhibitors (1∶100)) as previously described [31] 15, 30 or 60 min after angiopoietin treatment for Tie2 phosphorylation or 1, 5 or 15 min after thrombin stimulation for VE-cadherin phosphorylation. Tie2 phosphorylation was determined both in whole cell lysates and on immunoprecipitated Tie2 protein. VE-cadherin phosphorylation was determined in whole cell lysates. Tie2 antibody (goat, R&D systems) was used for immunoprecipitation as previously described [32]. In brief, cell lysates of equal amounts of protein in 1 ml of lysisbuffer as described above were incubated overnight at 4°C with the anti-human Tie2 (4 µg/sample) antibody (R&D systems). The protein-immunoglobulin G (IgG) complexes were pulled down using protein A/G agarose beads (Santa Cruz Biotechnology, Santa Cruz, USA) and after 3 washing steps laemmli buffer (Bio-Rad Laboratories, Hercules, California, USA) was added to the immunoprecipitated Tie2 protein. Total cell lysates containing equal amounts of protein or immunoprecipitated Tie2 protein from each sample were separated by SDS-PAGE and electrophoretically transferred to a nitrocellulose membrane (Bio-Rad Laboratories). Membranes were incubated with polyclonal antibodies against phosphorylated Tie2 (pTie2, Y1100, rabbit, 1∶333, R&D systems), phosphorylated tyrosine (pTyr, mouse, 1∶333, Cell signaling technology, Inc., Danvers, Massachusetts, USA), total Tie2 (tTie2, goat, 1∶500, R&D systems), phosphorylated VE-cadherin (tyrosine 685, rabbit, 1∶200, ECM Biosciences, Versailles, Kentucky, USA) or total VE-cadherin (rabbit, 1∶1000, Sigma). Total Tie2 and VE-cadherin were used as loading control. Goat anti-rabbit, goat anti-mouse and rabbit anti-goat immunoglobulins HRP (DakoCytomation, Glostrup, Denmark) were used for the detection of the primary antibodies at 1∶1000. Detection of the HRP reaction was performed with ECL plus Western Blotting Detection System (Amersham Biosciences, Little Chalfont Buckinghamshire, United Kingdom). Imaging and analysis were performed with LAS-3000 (Fuji Photo Film Co., Ltd., Tokyo, Japan) and AIDA Image Analyzer (Raytest GmbH, Straubenhardt, Germany). Data were expressed as phospho/total as fraction of control.

### Immunofluorescence

The cells were grown on gelatin-coated glass cover slips, treated for 30 min with angiopoietins (400 ng/ml) followed by thrombin stimulation (0.2 U/ml) for 2 and 15 min, fixed in 4% formaldehyde and permeabilized with 0.2% triton X-100. Cells were incubated with antibodies against VE-cadherin (1∶200, rabbit, overnight, 4°C, Sigma). Alexa fluor 488 goat anti-rabbit (1∶100, Invitrogen, Carlsbad, California, USA) was used for the detection of the primary VE-cadherin antibody. Actin filaments (F-actin) were stained with rhodamin-phalloidin (1∶100, Molecular Probes, Inc., Eugene, Oregon, USA). The nucleus was stained with 4′,6-diamidino-2-phenylindole (DAPI) in Vectashield® Mounting medium (Vector Laboratories, Inc., Burlingame, California, USA). Cells were visualized with help of fluorescence microscopy using a MarianasTM digital imaging microscope with 40X air lens (Carl Zeiss B.V., Sliedrecht, The Netherlands) and Slidebook 4.2 software (Intelligent Imaging Innovations, Inc., Denver, Colorado, USA).

To quantify the total gap area from VE-cadherin staining, so-called maskes were created as described previously [33], a mask being a binary overlay on an optical section. All VE-cadherin derived fluorescent signal below a threshold was included in a mask. The value for this threshold intensity was set manually below the baseline fluorescence intensity so as to select the total area negative for VE-cadherin, corresponding to the sites of inter-endothelial gaps. To exclude negative pixels that belonged to cellular regions, the individual fragments in the mask were gated to a minimal area of 50 pixels. Finally, the total area of all individual mask-fragments together was computed and expressed as a percentage of the total area of the field of view.

### G-LISA™ Rac1 or RhoA activation assay

The Rac1 and RhoA protein activity were measured with help of the G-LISA™ Rac1 or RhoA activation assay (Cytoskeleton, Inc., Denver, Colorado, USA) according to the manufacturers protocol. After one hour serum starvation with 1% HSA in EBM-2, cells were pre-incubated with Ang-2 (400 ng/ml), Ang-1 (400 ng/ml) or the combination for 30 min. After pre-incubation, thrombin (0.2 U/ml) was added and Rac1 or RhoA activity were measured 0, 1 or 15 min after addition of thrombin.

### Statistical analysis

Basal HRP passage, the HRP passage/hour and the decrease in TEER/min were not normally distributed (Kolmogorov-Smirnov test, P<0.05). To obtain normal distribution, data were logarithmically transformed prior to statistical analysis. A one-way analysis of variance (ANOVA) was conducted to explore the effects of the angiopoietins. The Student's t-test was used to further analyze the differences between the groups when ANOVA indicated statistical significance. Differences were considered significant at the P<0.05 level. Exact P-values are reported when P<0.05. Data are presented as mean ± standard error of the mean (SEM).

## Results

### Endogenous Ang-2 production

Under basal conditions, HPMVECs released Ang-2, but not Ang-1 in their medium at a constant rate of 139±14 pg/cm^2^/hour (P<0.0001) for at least 72 hours, so that after 48 hours ∼67 ng/ml Ang-2 was measured. To evaluate the effect of Ang-2, cells were serum-starved in fresh EBM-2 medium with 1% HSA one hour before the experiments, so that they were exposed to maximum ∼1.4 ng/ml endogenous Ang-2 before recombinant Ang-2 was administered. An Ang-2 concentration of 400 ng/ml was chosen for the experiments. Thrombin stimulation resulted in an release of as little as 0, 2.6 and 2.9 ng/ml endogenous Ang-2 at 1, 5 and 10 min after stimulation, respectively, suggesting a negligible effect of endogenous Ang-2 in the thrombin-induced permeability response. The detectable amount of Ang-2 in the conditioned medium decreased to 0.73 ng at 15 min after thrombin stimulation, suggesting degradation or binding of endogenous Ang-2.

### Basal effects of angiopoietins

**Figure 1:**
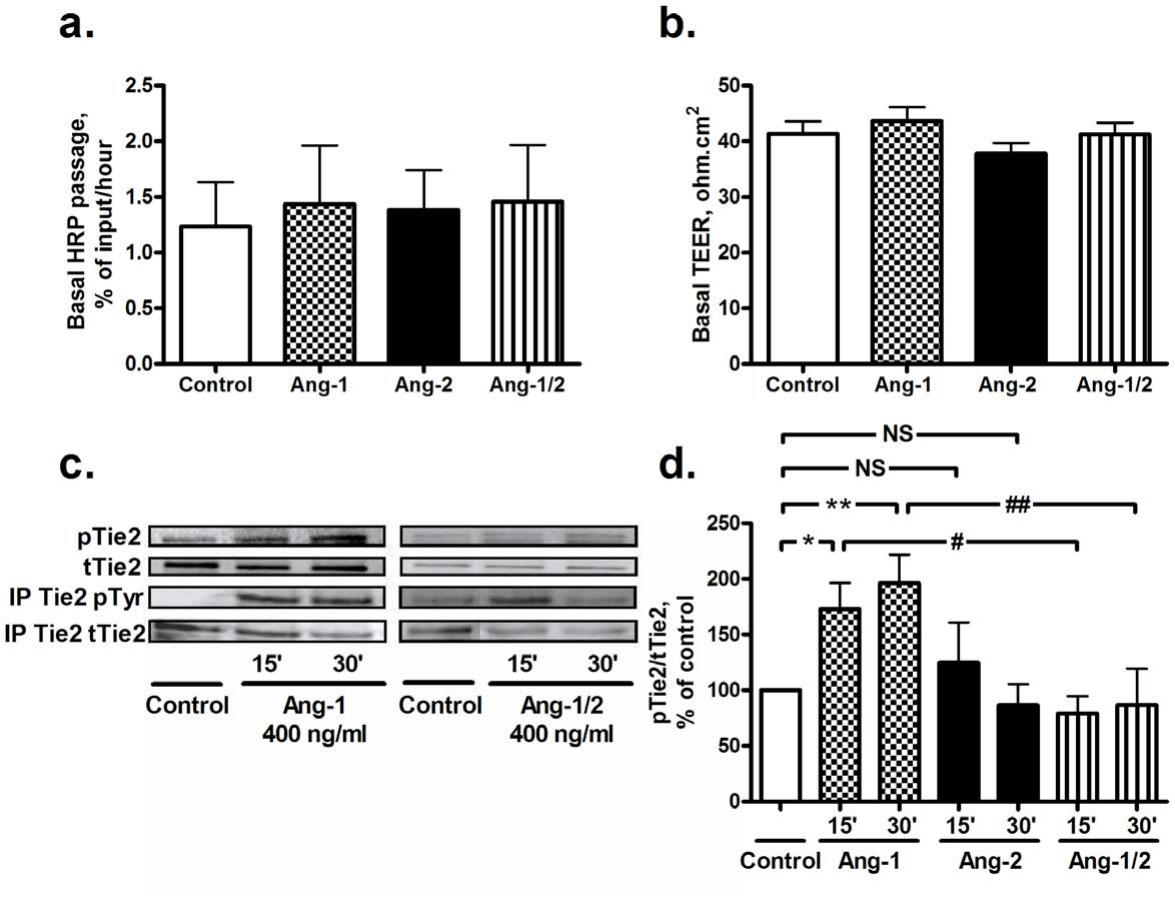
Effect of angiopoietins (Ang) on basal permeability, Tie2 phosphorylation and RhoA activity of human pulmonary microvascular endothelial cells (HPMVECs). Data are presented as mean ± standard error of the mean. NS: not significant. a. Neither Ang-2, nor Ang-1 affected the basal horse radish peroxidase (HRP) passage as measured 30 min after their addition (n = 7–9). b. Neither Ang-2, nor Ang-1 affected the basal transendothelial electrical resistance (TEER) as measured 30 min after their addition (n = 9–17). c. Representative western blots of pTie2, tTie and immunoprecipitated (IP) tyrosine phosphorylated (pTyr) and total Tie2 are shown. d. Ang-1 induced a transient increase in the phosphorylated Tie2 (pTie2)/total Tie2 (tTie2) ratio, with a maximum at 15 and 30 min after its addition (*P = 0.0046, **P = 0.0003). Ang-2 blocked the Ang-1-induced increase in Tie2 phosphorylation at 15 and 30 min (#P = 0.0073, ##P = 0.0134, n = 5–9). Ang-2 alone did not affect Y1100 Tie2 phosphorylation.

Unexpectedly, neither Ang-2, nor Ang-1 affected the basal endothelial barrier function as determined by HRP passage (Figure 1a) or TEER (Figure 1b). This was found with Ang-1 and -2 concentrations ranging from 5 to 400 ng/ml. To exclude that this was due to the short stimulation period (30 min), HPMVECs were stimulated with Ang-2 for 5 hours. Even after 5 hours, Ang-2 (5–400 ng/ml) did not affect the basal permeability as measured by the TEER (data not shown).

To confirm that the angiopoietins were able to activate the Tie2 receptor, Tie2 receptor phosphorylation was analyzed. Ang-1 induced a transient increase in Tie2 phosphorylation at the Y1100 tyrosine residue with a maximum increase at 15 and 30 min (Figure 1c and 1d). A similar pattern was observed for the total tyrosine phosphorylation of immunoprecipitated Tie2 (Figure 1c). Y1100 Tie2 phosphorylation returned to basal levels at 60 min (96±27 % of control). Co-treatment with Ang-2 blocked the Ang-1-induced Y1100 Tie2 phosphorylation at 15 and 30 min (Figure 1d). Ang-2 alone did not affect Y1100 Tie2 phosphorylation under our experimental conditions (Figure 1d).

Since RhoA and Rac1 are effectors of pTie2 [6], [13], their activity in HPMVECs under basal conditions 30 min after angiopoietin addition was measured. Ang-1 did not affect basal RhoA (93±10 % of control, n = 8) and enhanced basal Rac1 activity (123±7 % of control, P = 0.011, n = 6). Ang-2 did not affect basal RhoA (110±12 % of control, n = 8), but the trend was opposite to that of Ang-1. Ang-2 hardly affected Rac1 activity (108±3 % of control, P = 0.034, n = 6). Thrombin was used as a positive control. After 1 min exposure to thrombin RhoA activity was enhanced 3.7±0.3-fold (P<0.0001, n = 7), while Rac1 activity was reduced to 76±10 % of control (P = 0.0256, n = 5).

### Angiopoietin-induced modulation of the early thrombin-response

**Figure 2:**
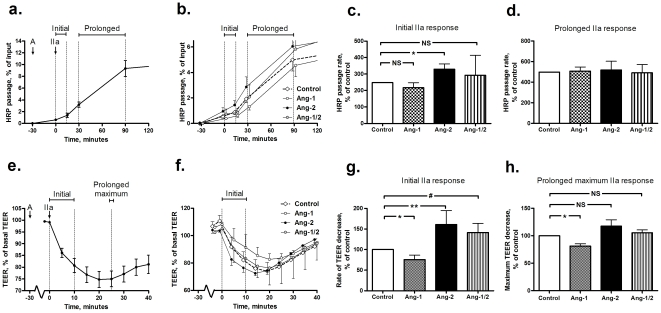
Effect of angiopoietins (Ang) on initial and prolonged permeability of human pulmonary microvascular endothelial cells (HPMVECs) during thrombin stimulation. Data are presented as mean ± standard error of the mean. NS: not significant. a. An averaged curve of horse radish peroxidase (HRP) passage of control cells stimulated with thrombin (IIa) at time = 0 min, showing the typical s-shape (n = 9). Cells were stimulated with angiopoietins (A) at time = −30 min. The initial HRP passage rate from time = 0–15 min and the prolonged HRP passage rate from time = 30–90 min were calculated as indicated by the vertical dashed lines. b. Representative experiment (in triplo) showing averaged curves of HRP passage of control and angiopoietin-stimulated cells. Cells were stimulated with angiopoietins at time = −30 min and with thrombin at time = 0 min. c. Ang-2 increased the initial HRP passage rate (*P = 0.010), while Ang-1 or the combination did not affect it (n = 7–9). d. Neither Ang-2, nor Ang-1 affected the prolonged HRP passage rate (n = 8–9). e. An averaged curve of the transendothelial electrical resistance (TEER) of control cells stimulated with thrombin at time = 0 min (n = 17). Cells were stimulated with angiopoietins at time = -30 min. The initial rate of TEER decrease from time = 0–10 min and the prolonged maximum TEER decrease were calculated as indicated by the vertical dashed lines. f. Representative experiment (in triplo) showing averaged curves of the TEER of control and angiopoietin-stimulated cells. Cells were stimulated with angiopoietins at time = −30 min and with thrombin at time = 0 min. g. Ang-2 and the combination enhanced the initial rate of the TEER decrease (**P = 0.021, ^#^P = 0.036), while Ang-1 reduced it (*P = 0.027, n = 12–16). h. Ang-2 or the combination did not affect the prolonged maximum TEER decrease, while Ang-1 reduced it (*P<0.0001, n = 12–16).

Subsequently, the effects of Ang-2 and Ang-1 on the thrombin-induced endothelial permeability were investigated. Differences in the effects of angiopoietin pre-treatment on the initial and the prolonged thrombin-induced endothelial permeability were observed (Figure 2a,e).

Ang-2 enhanced the initial thrombin-induced HRP passage rate from 0–15 min after thrombin addition, while Ang-1 or the combination of Ang-1 and Ang-2 did not affect it (Figure 2c). In contrast, neither Ang-2, nor Ang-1 affected the prolonged thrombin-induced HRP passage rate from 30–90 min (Figure 2d).

Since the TEER may be more sensitive to subtle thrombin-induced changes [28], [34], the effects of the angiopoietins on the thrombin-induced TEER decrease in both phases were also investigated (Figure 2f,g,h). The initial rate of the TEER decrease from 0–10 min after thrombin addition was enhanced by Ang-2 and the combination of Ang-1 and Ang-2 and reduced by Ang-1, a similar pattern as observed in the HRP passage experiments (Figure 2g). The prolonged maximum decrease in TEER was not affected by Ang-2 or the combination, while it was reduced by Ang-1 (Figure 2 h).

### Angiopoietins modulate VE-cadherin redistribution by thrombin

**Figure 3:**
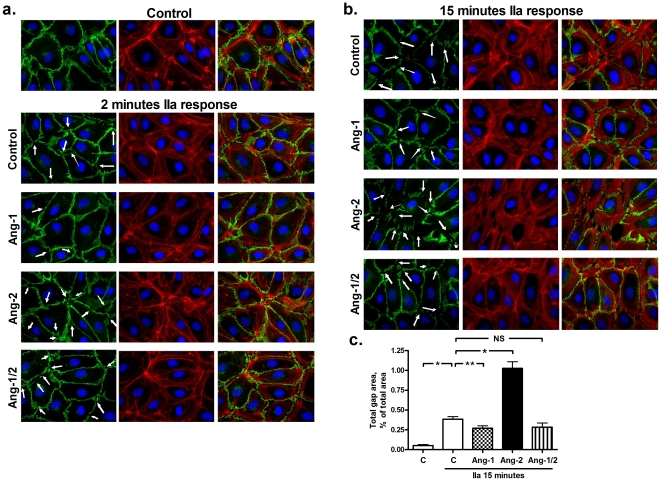
a, b and c. Effect of angiopoietins on the molecular organization of vascular endothelial cadherin (VE-cadherin) in human pulmonary microvascular endothelial cells (HPMVECs). Immunofluorescence morphological analysis and quantification of the distribution of VE-cadherin in control cells and cells treated with angiopoietins (Ang) and thrombin (IIa) for 2 (a) and 15 (b) min. Cells were stained with antibodies specific for VE-cadherin (green), with rhodamin-phalloidin for actin filaments (F-actin, red) and with 4′6-diamidino-2-phenylindole (DAPI) for the nuclei (blue). A 63x magnification is shown. The arrows indicate intercellular gaps. The left panel shows the VE-cadherin staining at the cell-cell borders. The middle panel shows the F-actin cytoskeleton. The right panel shows the merge of VE-cadherin, F-actin and the nuclei. a. The thrombin response after 2 min. b. The thrombin response after 15 min. c. Ang-2 enhances (*P<0.0001) and Ang-1 reduces (**P = 0.0257) the formation of thrombin-induced interendothelial gaps (*P<0.0001 thrombin stimulation vs. control). Total gap area was determined as described in the methods section. Data are the mean ± standard error of the mean from at least 6 pictures per condition.

The observation that the angiopoietins modulate in particular the initial response of endothelial cells to thrombin suggests that angiopoietins may affect the molecular organization of the adherence junctions [35]. To study the molecular organization of the adherence junctions, VE-cadherin was visualized by immunofluorescence microscopy as shown at 2 and 15 min after thrombin stimulation in Figures 3a and b, respectively. VE-cadherin was encountered in control cells as a continuous and narrow lining at cell-cell borders reflecting stable junctions. Exposure to thrombin induced a redistribution of VE-cadherin into a zigzag wide pattern typical for unstable and activated junctions and the generation of intercellular gaps. Ang-1 preincubation attenuated the effect of thrombin on VE-cadherin redistribution and gap formation. However, still some intercellular gaps were visible in accordance with the thrombin-induced permeability in Ang-1 treated cells (Figure 3c). In contrast, the effect of thrombin was enhanced in Ang-2 treated cells, which resulted in even wider VE-cadherin staining and a stronger zigzag pattern together with more intercellular gaps (Figure 3c). Ang-1 prevented the enhancement of gap formation in Ang-2 treated cells (Figure 3c). The response at 2 and 15 min was similar, although the thrombin-induced gaps were more pronounced at 15 min.

The changes in VE-cadherin were accompanied by alterations in the F-actin cytoskeleton. While in control cells most F-actin bundles were seen in the periphery of the cell, thrombin induced the formation of F-actin bundles throughout the cell. However, although there was a counteracting tendency by the presence of Ang-1, the effects of Ang-1 and Ang-2 on thrombin-stimulated stress fiber formation were very limited (Figure 3a and b).

We subsequently evaluated tyrosine phosphorylation of VE-cadherin [18], [35], with specific emphasis on that of the tyrosine 685 residue, since this residue is phosporylated by Src [35], which is linked to angiopoietin signaling in the context of vascular endothelial growth factor (VEGF)-induced endothelial permeability [36]. VE-cadherin phosphorylation at tyrosine 685 was not affected by thrombin stimulation, independent of angiopoietin treatment (supporting information Figure S2).

### Angiopoietins and RhoA and Rac1 activity in thrombin stimulated cells

**Figure 4:**
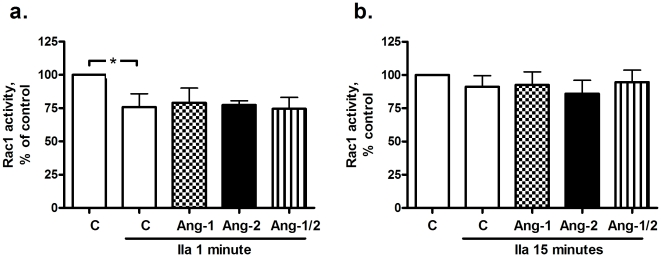
Effect of angiopoietins (Ang) on Rac1 activity of thrombin (IIa)-stimulated human pulmonary microvascular endothelial cells (HPMVECs). Data are presented as mean ± standard error of the mean. a. Thrombin stimulation reduced Rac1 activity 1 min after stimulation (*P = 0.0256), independent of angiopoietin treatment (n = 5–6). b. Rac1 activity was normalized 15 min after thrombin stimulation, independent of angiopoietin treatment (n = 5–6).

**Figure 5:**
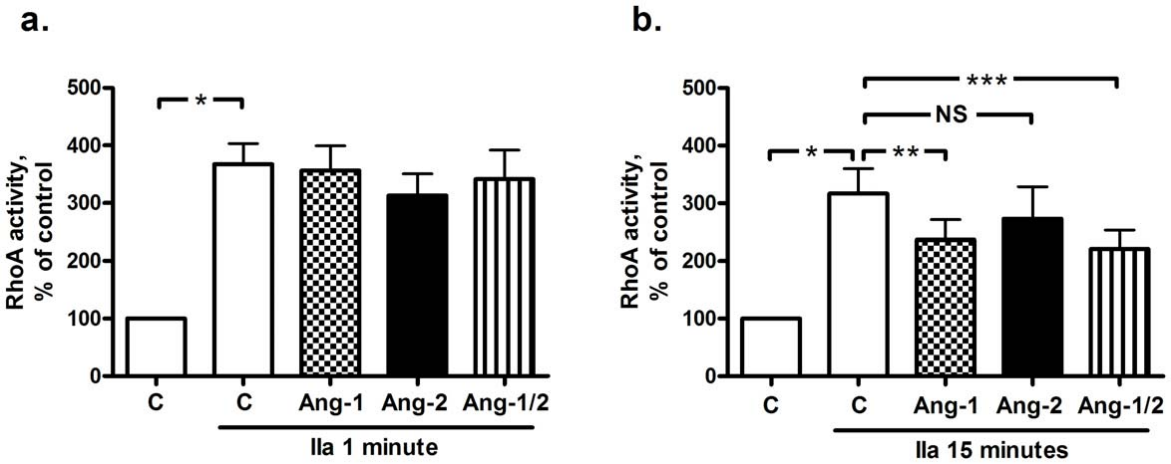
Effect of angiopoietins (Ang) on RhoA activity of thrombin (IIa)-stimulated human pulmonary microvascular endothelial cells (HPMVECs). Data are presented as mean ± standard error of the mean. NS: not significant. a. RhoA activity was increased 1 min after thrombin addition (*P<0.0001), independent of angiopoietin treatment (n = 7–8). b. RhoA activity was still increased 15 min after thrombin addition (*P<0.0001), but the increase became less in cells treated with Ang-1 (**P = 0.039, n = 6) or the combination (***P = 0.034, n = 6-8).

Disruption of the adherence junctions between cells may be amongst others due to reduced Rac1 activity [25], and indirectly influenced by RhoA-mediated actin-myosin interaction [26]. Since both small GTPAses are described as effectors downstream of Tie2 phosphorylation [5], [12], Rac1 and RhoA activities were determined at various time points ranging from 1 to 15 min after thrombin addition. Rac1 activity was decreased at 1 min after thrombin stimulation (Figure 4a) and normalized after 15 min (Figure 4b), independently of the presence of angiopoietins. In contrast, thrombin induced a 3- to 4-fold increase in RhoA activity after 1 min compared to control (Figure 5a), which gradually decreased to 3-fold after 15 min (Figure 5b). While the increase of RhoA after 1 min was not altered by the presence of angiopoietins (Figure 5a), RhoA activity at 15 min decreased more rapidly when Ang-1 or the combination were present. This effect was absent in cells treated with Ang-2 (Figure 5b).

## Discussion

The main finding of the present study is that the angiopoietins Ang-2 and Ang-1 had opposing effects on the very initial, but not on the prolonged late phase of the thrombin-induced hyperpermeability response of cultured human pulmonary microvascular endothelial cells. Specifically, Ang-2 enhanced the initial hyperpermeability, while Ang-1 reduced the initial hyperpermeability by attenuation of thrombin-induced reorganization of the adherence junctions. The limited effect of angiopoietins on basal permeability as compared to thrombin-stimulated permeability suggests that in the adult endothelium the angiopoietin-Tie2 system is a sensitizer of the activated endothelium in the presence of other inflammatory or coagulation mediators, rather than an independent actor of the permeability response. This is in line with previous findings that Ang-2 alone did not affect the adhesion of leukocytes to quiescent endothelium, while it promoted adhesion of leukocytes to endothelial cells activated by tumor necrosis factor-α [10].

The permeability enhancing effect of thrombin in endothelial monolayers in culture can be separated in two phases. During the initial phase (0–15 min) the TEER decreases rapidly, while the passage of HRP starts increasing. During this phase the endothelial junctions become instable and locally small gaps between the cells are formed. After 15 min stress fibers have become formed reflecting a major change in the F-actin-cytoskeleton. In the subsequent phase (15–90 min) the rate of HRP passage becomes maximal. This phase includes continued actin-myosin interaction within the cells and cell contraction [16], [37]. However, the TEER starts to recover during this period suggesting that junctional complexes and focal adhesion sites are locally recovering, although still relatively large gaps between cells remain. After 90 min a full recovery of the monolayer is observed both with regard to TEER and HRP passage.

In the context of this dual effect of thrombin, the effect of angiopoietins only on the initial thrombin response is of interest and points to an effect at the junctional level in particular. Indeed, we observed changes in VE-cadherin localization that reflected unstable junctions and intracellular gap formation. While similar alterations in adherence junctions and VE-cadherin relocalization are induced by VEGF via Src phosphorylation at Tyr685 and subsequent activities [35], thrombin did not affect this phosphorylation. This is accordance with Kinney et al. [38], who showed that thrombin has no effect on Src and Yes, but only on the Src-like protein Fyn, which has less permeability enhancing properties [39]. Apparently another mechanism induces the dissociation of VE-cadherins in adherence junctions.

Notwithstanding, our data support previous findings that Ang-1 inhibits the thrombin response by enforcement of junctions via enforcement of the VE-cadherin-catenin complex [19], similar as observed in VEGF- and bradykinin-induced hyperpermeability [36], [40]–[42]. After exposure of human endothelial cell monolayers to Ang-1, Tie-2 receptors are mobilized from the endothelial cell surface to the cell junctions, where oligo- or multimers of Ang-1 [43] bridge Tie-2 receptors of both adjacent cells [41], [44]. This complex also recruits vascular endothelial protein tyrosine phosphatase (VE-PTP) [45]. At these junctions the multimeric complex of Ang-1 and Tie-2 bridges two cells [41], [44] and induces specific Tie-2-mediated signaling that causes activation of small GTPase Rap1 and subsequently Rac1, which enforce the maintenance of the junctions between both cells [46]–[48]. Such mechanism underlies the protective effect of Ang-1 on VEGF-induced hyperpermeability [36], [40], [45] and on the initial thrombin induced hyperpermeability as presently and previously observed [19]–[21].

Several additional signaling mechanisms have been reported, namely Ang-1 inhibited the thrombin response by reduction of the cytoplasmic calcium concentration [49] or PKC-æ activity [21], [50]. In addition, Mammoto et al. [5] pointed towards an increased activity of the inhibitory GTPase activating protein p190 RhoGAP as a contributor to the inhibitory effect of Ang-1 on endotoxin-mediated vascular leakage. As thrombin induces RhoA activity, a similar mechanism may contribute to the effects observed in the present HPMVECs. Activation of p190RhoGAP by Ang-1 limits the activation of Rho kinase and mDia, which can affect subsequent pathways that enhance permeability [5], [36]. Indeed, Ang-1 caused a reduction in RhoA activation when assayed 15 min after thrombin stimulation, conform Mammoto et al. [5], but not at earlier time points (see also [20]). Therefore, modulation of RhoA activity becomes in particular important when the junctions were already destabilized by the initial response.

To our knowledge, we are the first to demonstrate that Ang-2 enhanced thrombin-induced endothelial permeability in HPMVECs, similar to the effect of Ang-2 on VEGF-induced retinal endothelial cell permeability [51]. Interestingly, Ang-2 enhanced the initial permeability in particular, suggesting that Ang-2 modulates the stability of the junctions before or during the initial rapid increase in thrombin-induced permeability [16], [18], but has less effect during the later phase of the cell contraction after formation of stress fibers, i.e. when the junctional multimeric Ang-1/Tie-2 complexes had disappeared. Indeed, Ang-2 induced a change in the molecular organization of the junctions as demonstrated by an enhancement of the zigzag pattern, while it did not enhance the number or organization of stress fibers during thrombin stimulation. Ang-2 did not enhance VE-cadherin phosphorylation at tyrosine 685, as seen in other conditions [35]. However, the availability of Tyr685 depends on Csk binding [52], while other VE-cadherin tyrosine residues may be phosphorylated by Ang-2 [35]. Alternatively, Ang-2 may act by preventing protective actions on adherence junction proteins. In line with this suggestion, Seegar et al. [53] reported that Ang-2 enhances Tie-1-Tie-2 interaction, which inhibits the endothelial protective effect of Tie-2 activation. This in contrast to Ang-1, which directs protective Tie-2 activity by homomultimerization [53]. This latter action of Ang-1 probably also explains why the combination of equal concentrations of Ang-1 and Ang-2, which in most studies have equal affinities for the Tie2 receptor [54]–[56], still enhanced the initial rate of the thrombin-induced permeability, albeit slightly less than Ang-2 alone.

Whether the withdrawal of Tie-2 from junctional multimerization also causes the increase in thrombin-induced hyperpermeability when only Ang-2 is added, is uncertain, because endothelial cells produce little Ang-1 themselves [57]. Signaling by direct interaction of Ang-2 with Tie-1 into the endothelial cell has also been reported [58] and may affect junction stability in thrombin-stimulated cells. Finally, Ang-2 can activate endothelial cells via other phosphorylation sites on the Tie2 receptor [32], while the interaction between the F-actin cytoskeleton and junctional proteins may also be affected. RhoA is an important mediator of thrombin-induced actin-myosin interaction, which also causes stress fiber formation and cell contraction. Parikh et al. [12] reported that Ang-2 increased basal permeability via increased RhoA activity, but did not study the effect of Ang-2 on thrombin-induced RhoA activity. In our experiments Ang-2 did not affect the degree of RhoA activation at 15 min after thrombin-stimulation or under basal conditions. However, it should be noted that Parikh et al. [12] observed an unusual prolonged increased RhoA activity up to 6 hours, which suggests that an additional activation of the HPMVECs has occurred that affected their responsiveness to Ang-2 [10].

The responses to Ang-1 and Ang-2 were relatively small. We cannot not exclude that the HPMVECs had reduced sensitivity for Ang-2, due to the endogenous production of Ang-2 by endothelial cells themselves. Nevertheless, cells were stimulated with approximately 6 times higher concentrations of Ang-2 than they encountered normally during culture. Furthermore, it should be noted that thrombin-induced macromolecule passage largely takes place via the paracellular pathway through intercellular gaps and depends on the molecular size of the macromolecule [28]-[30]. The present data cannot completely exclude additional effects of the angiopoietins on transcellular exchange.

In conclusion, the present study describes the effects of angiopoietins related to the kinetics of the thrombin-response in HPMVECs. The effects of angiopoietins are only found in the initial junction-related thrombin-induced permeability and not in the prolonged stress fiber contraction-related phase of thrombin-induced permeability. As the latter phase is accompanied by abundant stress fiber formation to an extent that is normally not seen in vivo, it is likely that the initial phase provides a better reflection of pathophysiological alterations of the endothelial barrier. This fits with the current knowledge of the effect of Ang-1, and our data add new information regarding a potential role of Ang-2.

## Supporting Information

Figure S1 **Characterization of cultured human pulmonary microvascular endothelial cells (HPMVECs).** Phase-contrast pictures of HPMVECs 5 and 9 days after isolation are shown. Subsequent panels show representative fluorescent images of vascular endothelial (VE)-cadherin, CD31, von Willebrand factor (VWF), smooth muscle cell (SMC) α-actin and pancytokeratin and representative western blots of Tie2 and endothelial nitric oxide synthase (eNOS).
(TIF)

Figure S2 **Angiopoietins do not affect vascular endothelial cadherin (VE-cadherin) phosphorylation of human pulmonary microvascular endothelial cells (HPMVECs).** Representative western blots of VE-cadherin phosphorylated (p) at tyrosine residue 685 and total VE-cadherin in control (C), Ang-1 (A1) and Ang-2 (A2) treated cells as measured 1, 5 and 15 min after thrombin (IIa) addition.
(TIF)

Text S1 (DOC)
