# Supplementary figures and images for: Opposing Effects of the Angiopoietins on the Thrombin-Induced Permeability of Human Pulmonary Microvascular Endothelial Cells
Source: PLoS One. 2011 Aug 15;6(8):e23448. doi: 10.1371/journal.pone.0023448 (PMC3156229; doi:10.1371/journal.pone.0023448)

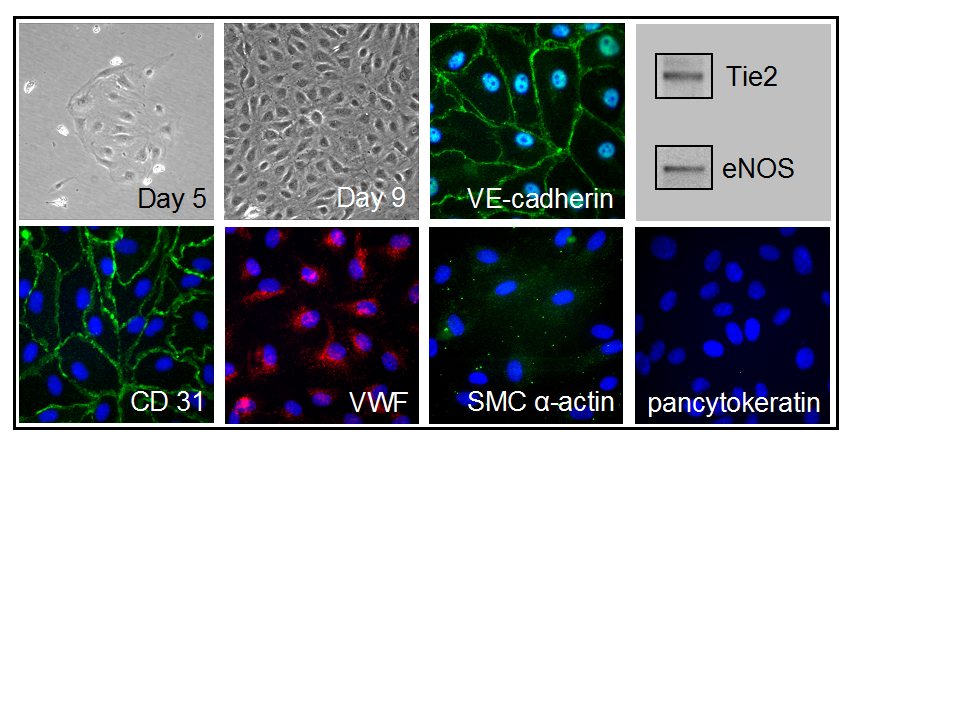

Supplement: Figure S1 — Characterization of cultured human pulmonary microvascular endothelial cells (HPMVECs). Phase-contrast pictures of HPMVECs 5 and 9 days after isolation are shown. Subsequent panels show representative fluorescent images of vascular endothelial (VE)-cadherin, CD31, von Willebrand factor (VWF), smooth muscle cell (SMC) α-actin and pancytokeratin and representative western blots of Tie2 and endothelial nitric oxide synthase (eNOS). (TIF) [file pone.0023448.s001.tif]

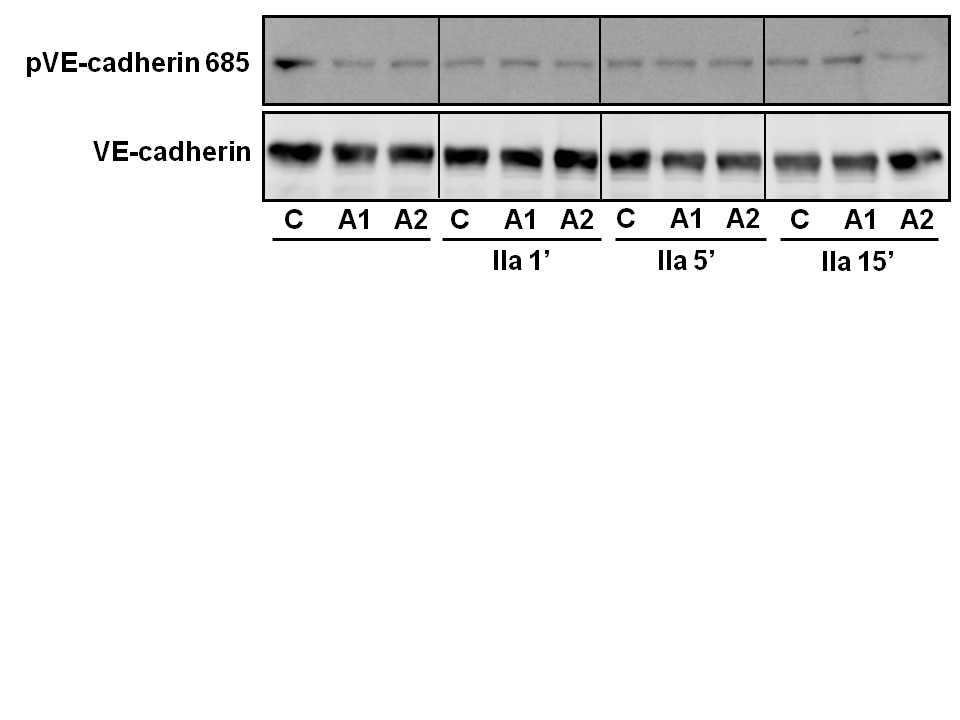

Supplement: Figure S2 — Angiopoietins do not affect vascular endothelial cadherin (VE-cadherin) phosphorylation of human pulmonary microvascular endothelial cells (HPMVECs). Representative western blots of VE-cadherin phosphorylated (p) at tyrosine residue 685 and total VE-cadherin in control (C), Ang-1 (A1) and Ang-2 (A2) treated cells as measured 1, 5 and 15 min after thrombin (IIa) addition. (TIF) [file pone.0023448.s002.tif]
