# Supplementary material for: Opposing Effects of the Angiopoietins on the Thrombin-Induced Permeability of Human Pulmonary Microvascular Endothelial Cells
Source: PLoS One. 2011 Aug 15;6(8):e23448. doi: 10.1371/journal.pone.0023448 (PMC3156229; doi:10.1371/journal.pone.0023448)
Supplement: Text S1 — (DOC) [file pone.0023448.s003.doc]

**SUPPORTING INFORMATION TEXT S1**

**Isolation and culture of HPMVECs**

HPMVECs were isolated as previously described [1,2]. Briefly, after lobectomy for carcinoid or metastasis, approximately 1 cm^3^ of healthy peripheral human lung tissue from a normal appearing region farthest away from the tumor was isolated. The lung tissue was rinsed in 10 mM phosphate-buffered saline (PBS, pH 7.4), separated from the overlying pleura and cut into 2-3 mm^3^ pieces with scissors. The minced tissue was digested in 20 ml 0.3% collagenase type 2 (Sigma, St. Louis, Missouri, USA) in PBS for 1 hour at 37 °C with occasional agitation. The digestion was stopped by addition of 6 ml of microvascular endothelial cell medium-2 (EGM-2-MV, Lonza, Basel, Switzerland). The digested suspension was filtered through a 100 μm strainer (Falcon, Franklin Lakes, New Jersey, USA) and centrifuged for 5 min at 500xg. The cell pellet was resuspended in binding buffer (2 μM Na-citrate, 1.2 μM NaH_2_PO_4_∙H_2_O, 5.6 μM Na_2_HPO_4_, 138.6 μM NaCl, 0.1% bovine serum albumin), filtered through a 70 μm strainer (Falcon) and centrifuged for 5 min at 500xg. The cell pellet was resuspended in 1 ml EGM-2-MV medium and centrifuged for 5 min at 500xg. The cell pellet was resuspended in 120 μl EGM-2-MV medium, 40 μl FcR blocking reagent (Miltenyi Biotec GmbH, Bergisch Gladbach, Germany) and 40 μl magnetic microbeads coated with anti-human CD31 antibody (Miltenyi Biotec) and incubated for 15 min at 4 ºC in a rotator. 1 ml of EGM-2-MV was added to the cell suspension and the suspension was centrifuged for 5 min at 500xg. The cell pellet was resuspended in 3 ml EGM-2-MV medium and transferred to a LS column in the magnetic field of a MidiMACS Separator (Miltenyi). The column was washed three times with 3 ml of EGM-2-MV medium to remove unlabeled cells. The column was removed from the magnetic field, placed on a collection tube and the magnetically labeled CD31+ cells were flushed out. Isolated cells were resuspended in 10 ml EGM-2-MV medium with 10% fetal bovine serum and cultured at 37 °C under 5% CO_2_/95% air atmosphere in a fibronectin-coated 75 cm^2^ cell culture flask. Culture medium was renewed the following day to remove unattached cells, and the medium was subsequently changed every other day. Ten days after seeding the cells, cells were harvested with trypsin/ethyleendiaminetetraacetic acid (EDTA), and the magnetic separation was repeated as described above. Cells were subsequently cultured in EGM-2-MV medium with 5% fetal bovine serum.

**Characterization of HPMVEC culture**

To characterize the HPMVEC culture, the presence of endothelial cell markers and the absence of smooth muscle cell (SMC) and epithelial cell markers were evaluated. The cells were grown on gelatin-coated glass cover slips, fixed in 4% formaldehyde and permeabilized with 0.2% triton X-100. Cells were incubated with antibodies against vascular endothelial cadherin (VE-cadherin, rabbit, 1:100, overnight, 4 ºC, Sigma), CD31 (mouse, 1:40, overnight, 4 ºC, BD Pharmingen, Franklin Lakes, New Jersey, USA), von Willebrand factor (VWF, sheep, 1:40, overnight, 4 ºC, Santa Cruz Biotechnology, Inc., Santa Cruz, California, USA), SMC marker α-actin (mouse, 1:50, overnight, 4 ºC, DakoCytomation, Glostrup, Denmark) and epithelial cell marker pancytokeratin (mouse, 1:50, overnight, 4 ºC, DakoCytomation). The nucleus was stained with 4',6-diamidino-2-phenylindole (DAPI) in Vectashield® Mounting medium (Vector Laboratories, Inc., Burlingame, California, USA). Rabbit anti-mouse fluorescein isothiocyanate (FITC, 1:30, DakoCytomation), alexa fluor 488 goat anti-rabbit (1:100, Invitrogen, Carlsbad, California, USA) and donkey anti-sheep Cy5 (1:30, Jackson ImmunoResearch Laboratories, Inc., West Grove, Pennsylvania, USA) were used for the detection of the primary antibodies with help of fluorescence microscopy using a Marianas^TM^ digital imaging microscope with 40X air lens (Carl Zeiss B.V., Sliedrecht, The Netherlands) and Slidebook 4.2 software (Intelligent Imaging Innovations, Inc., Denver, Colorado, USA).

To further characterize the HPMVEC culture, the endothelial specific proteins Tie2 and endothelial nitric oxide synthase (eNOS) were analyzed with help of western blotting. After serum starvation, cells were lysed and equal amounts of protein from each sample were separated as previously described [3]. Polyclonal antibodies against total Tie2 (tTie2, goat, 1:500, R&D systems, Minneapolis, Minneapolis, USA) and eNOS (rabbit, 1:1000, Santa Cruz Biotechnology, Inc.) were used. Rabbit anti-goat and goat anti-rabbit immunoglobulins horse radish peroxidase (HRP) (DakoCytomation) were used for the detection of the primary antibodies at 1:1000. Detection of the HRP reaction was performed as previously described [3].

1. Shelton JL, Wang L, Cepinskas G, Sandig M, Inculet R, et al. (2006) Albumin leak across human pulmonary microvascular vs. umbilical vein endothelial cells under septic conditions. Microvasc Res 71: 40-47.
2. Wagner M, Hermanns I, Bittinger F, Kirkpatrick CJ (1999) Induction of stress proteins in human endothelial cells by heavy metal ions and heat shock. Am J Physiol 277: L1026-L1033.
3. Van der Heijden M, Versteilen AM, Sipkema P, Van Nieuw Amerongen GP, Musters RJ et al. (2008) Rho-kinase-dependent F-actin rearrangement is involved in the inhibition of PI3-kinase/Akt during ischemia-reperfusion-induced endothelial cell apoptosis. Apoptosis 13: 404-412.
